# Simultaneous Determination of Aflatoxins and Benzo(a)pyrene in Vegetable Oils Using Humic Acid-Bonded Silica SPE HPLC–PHRED–FLD

**DOI:** 10.3390/toxins14050352

**Published:** 2022-05-18

**Authors:** Di Yuan, Liangxiao Zhang, Fei Ma, Peiwu Li

**Affiliations:** 1 Oil Crops Research Institute, Chinese Academy of Agricultural Sciences, Wuhan 430062, China; yuandi199707@163.com (D.Y.); zhanglx@caas.cn (L.Z.); peiwuli@oilcrops.cn (P.L.); 2 Key Laboratory of Biology and Genetic Improvement of Oil Crops, Agriculture and Rural Affairs, Wuhan 430062, China; 3 National Reference Laboratory for Agricultural Testing (Biotoxin), Wuhan 430062, China; 4 Key Laboratory of Detection for Mycotoxins, Wuhan 430062, China; 5 Laboratory of Quality & Safety Risk Assessment for Oilseed Products (Wuhan), Wuhan 430062, China

**Keywords:** aflatoxins, solid-phase extraction (SPE), quantification, high-performance liquid chromatography with photochemical post-column reactors fluorescence detector (HPLC–PHRED–FLD), vegetable oil

## Abstract

In the present work, a rapid, accurate, and cost-effective method was developed for the simultaneous quantification of aflatoxins and benzo(a)pyrene in lipid matrices, using solid-phase extraction (SPE) via humic acid-bonded silica (HAS) sorbents, followed by high-performance liquid chromatography coupled with photochemical post-column reactor fluorescence spectroscopy (HPLC–PHRED–FLD) analysis. The major parameters of extraction efficiency and HPLC–PHRED–FLD analysis were investigated and this method was fully validated. The limits of quantification and the limits of detection were 0.05–0.30 and 0.01–0.09 µg kg^−1^, respectively. The recoveries were 66.9%–118.4% with intra-day and inter-day precision less than 7.2%. The results of 80 oil samples from supermarkets indicated a high occurrence of BaP, and most of concentrations were within the requirements of EU and China food safety regulations. This is the first utilization of HAS–SPE HPLC–PHRED–FLD to simultaneously analyze the occurrence of aflatoxins and benzo(a)pyrene in vegetable oils.

## 1. Introduction

Vegetable oils provide calories, essential fatty acids, and functional compounds in the human diet, and also transport fat-soluble nutrients. Oilseeds, especially soybean, rapeseed, peanut, and sunflower, are important fatty acid sources that are often subjected to heat treatment to increase oil production and improve sensory quality [1]. Processing technology have been applied to improve the physical and chemical characteristics of vegetable oils, and these changes are related to the raw material, as well as the heat and refinement treatment used [2]. Storage and heating affect the quality and safety of oilseed, and these processes can also result in the formation and transference of hazardous compounds into vegetable oils [3].

Many studies have reported mycotoxins and polycyclic aromatic hydrocarbons found in vegetable oils, due to fungal infection and high-temperature pyrolysis. Among those toxic molecules, aflatoxins (AFs) and benzo(a)pyrene (BaP) are widely known contaminants with great economic and health impacts [4,5]. They have been identified in oilseed-based products, especially related to vegetable oils. Both AFs and BaP can cause cytotoxicity, immune toxicity, teratogenicity, and carcinogenicity in humans and animals [6,7]. The International Agency of Research on Cancer (IARC) classified AFB_1_ and BaP as group I carcinogens in 1993 and 2010 [8,9]. Consequently, many countries and international organizations have set strict maximum residue limits (MRLs) to monitor and control co-contaminations in foods. For aflatoxins, the United States established the MRL for the sum of AFs in all foods at 20 μg kg^−1^, and China established the MRLs for aflatoxin B_1_ (AFB_1_) in peanut, corn, and the other vegetable oils at 20 and 10 μg kg^−1^, respectively. For BaP, European Commission (EU) set the MRL for BaP in edible oil at 2 μg kg^−1^, and China established the MRL for BaP in vegetable oils at 10 μg kg^−1^ [10,11]. Therefore, it is vital to develop a simple and accurate method to monitor and evaluate the co-contamination of AFs and BaP in vegetable oils.

Currently, various instrumental methods are being used to quantify AFs and BaP in food matrices, including enzyme-linked immunosorbent assays [12], nanogold probe-based immunochromatographic assay [13], and high-performance liquid chromatography coupled with mass spectrometry (MS)/fluorescence detectors [14,15]. Owing to its simplicity, feasibility, and accuracy, high-performance liquid chromatography with fluorescence detector (HPLC–FLD) has been widely adopted as the standard method of AFs determination in vegetable oils. Additionally, the post-column online photochemical system coupled with HPLC–FLD has gained widespread popularity, as it offers enhanced sensitivity and accuracy as well as MS detector, and avoids the application of toxin reagents and complex derivatization steps, which are specific for analytes which are difficult to turn charged ions with electrospray ionization or atmospheric pressure chemical ionization.

At present, many pretreatment procedures for the extraction and purification of AFs or BaP from lipid matrices have been established, including liquid–liquid extraction (LLE) [16], dispersive liquid–liquid microextraction (DLLME) [17], molecular imprinting (MIP) [18], magnetic solid-phase extraction (MSPE) [19], and immunoaffinity column (IAC) purification [20]. Most of these pretreatments are complicated, tedious, and require large volumes of organic solvents and sophisticated apparatus for adsorbent preparation, centrifugation, and extraction. Therefore, a simple, rapid and efficiency pretreatment method is important to determine those analytes in lipid matrice.

Solid-phase extraction (SPE) is widely acknowledged and applied to isolate and purify analytes in concentrations as low as μg kg^−1^ from environmental, biomedical, and food matrices in controlled laboratory settings. SPE is an effective method to preconcentrate and purify the extracts by partitioning the analytes between the solid adsorbents and liquid solutions. This technique effectively and selectively adsorbs the target analyte with automated procedures for large-scale food quality and safety surveys [21]. The classic SPE adsorbents include silica, carbon, and polymeric materials based on the functional group C_18_ [22], Oasis HLB [23], as well as graphite carbon black (GCB) [24]. Humic acids (HAS) were the natural substances produced by the degradation of animal and plant residues by microorganisms. HAS materials have been proven to contain alkyl and aromatic rings, as well as carboxylic acid, phenolic hydroxyl, quinone, and amino functional groups [25]. These substituents facilitate chelation with metal ions, oxides, and some toxic substances [26]. HAS is an effective SPE adsorbent for the extraction and purification of BaP or AFs. Humic acid-bonded silica material had been prepared and used as SPE adsorbent for enrichment and purification of BaP in edible vegetable oils coupled with HPLC–FLD analysis [27]. A low-cost and effective method had been proposed using SPE HPLC–MS/MS for the determination of AFs in edible oils [28].

With time-consuming steps and sophisticated apparatus, few studies have reported the simultaneous determination of AFs and BaP by gel permeation chromatography coupled with liquid chromatography-fluorescence [29]. Therefore, the aim of this work was to develop a simple, rapid, and cost-effective quantification method using humic acid-bonded SPE coupled with HPLC–PHRED–FLD to determine AFs and BaP contamination in vegetable oil.

## 2. Results and Discussion

### 2.1. Optimization of HPLC–PHRED–FLD

**Figure 1:**
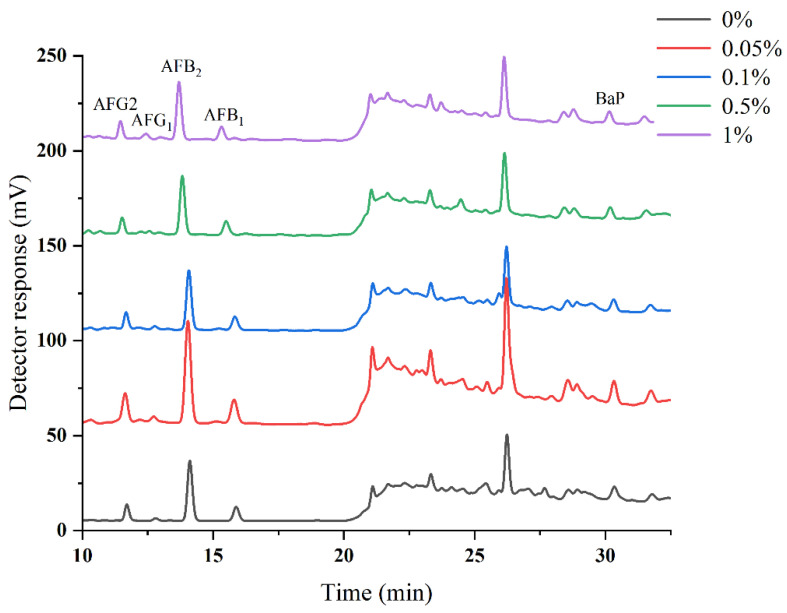
Chromatograms of AFs and BaP with different concentrations of formic acid as the mobile phase.

The molecular diversity and lipophilicity characteristics of the investigated compounds play an important role during HPLC analysis. The lipophilicity of the analytes with log *p* values ranged from 1.63 to 6.39; thus, the mobile phase mainly consisted of organic solvent and water under neutral or acidic conditions. A mobile-phase system consisting of methanol–aqueous formic acid was selected to achieve good separation with acceptable sensitivity of the FLD detector. Moreover, methanol has lower toxicity than acetonitrile. As shown in Figure 1, the HPLC chromatographic peaks of AFB_1_ and BaP increased dramatically as the content of formic acid increased from 0% to 0.05%, and the peaks gradually decreased as the content of formic acid increased continuously from 0.05% to 1%. Therefore, methanol and aqueous 0.05% formic acid were employed as the mobile phase in the following gradient elution procedure.

**Figure 2:**
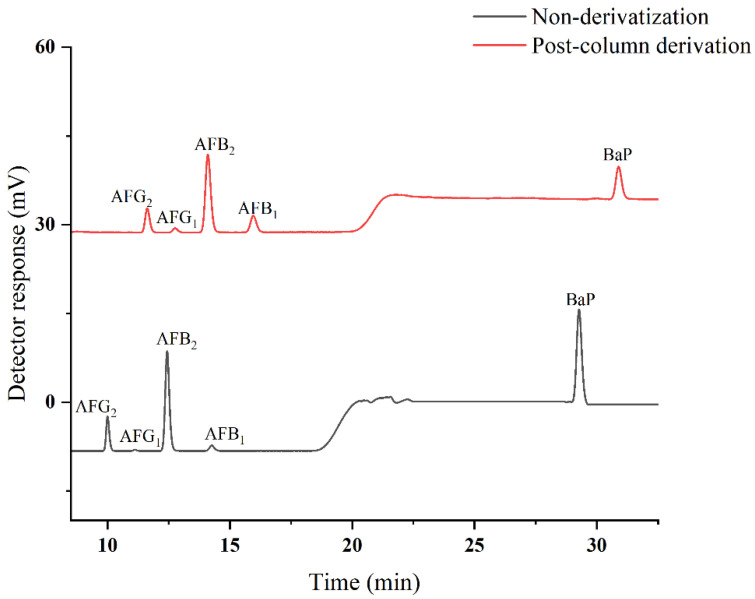
Pre-column chromatograms of derivatized and non-derivatized AFs and BaP.

In previous works on HPLC–FLD detection, excitation and emission wavelengths (λ_ex_/λ_em_) for AFs and BaP were 360/440 and 384/406 nm, respectively. Due to variation in molecular structures and chemical properties, excitation and emission wavelengths, as well as fluorescence spectra of AFs and BaP, should be further improved. Specifically, the requirement of an online post-column photolysis PHRED system for AFs should be reconsidered. As depicted in Figure 2, post-column derivatization prolongs the overall analysis times. However, it significantly increased the response value of AFB_1_ and AFG_1_ and only slightly reduced the response of BaP in an acceptable range. Under the wavelengths of λ_ex_/λ_em_ at 380/420 nm, high sensitivity and baseline separation were achieved for AFB_1_, AFB_2_, AFG_1_, AFG_2_, and BaP in approximately 30 min, without interference.

### 2.2. Optimization of HAS Solid-Phase Extraction

The HAS–SPE procedure was optimized using blank soybean oil with 5.0 μg kg^−1^ AFs and BaP, and the SPE column was packed with 500 mg adsorbents. Different parameters, including SPE materials, loading solvent, washing solvent, and the type and volume of eluting solvent, were studied accordingly.

#### 2.2.1. Loading Solvent

Vegetable oils are viscous mixtures of glycidyl esters and lipid-soluble compounds, and the pH values range from 3.2 to 6.0, which are below the pKa values of AFs and BaP [30]. To improve the viscosity and retain pH-dependent analytes in the extraction, the oil sample was diluted with nonpolar *n*-hexane (1/4, *v*/*v*). All of the analytes were retained by the HAS adsorbents, and polar interferences passed through the SPE column with the diluted extracts at a flow rate of 2 mL min^−1^. Therefore, 8 mL of *n*-hexane was used to dilute the oil.

#### 2.2.2. SPE Adsorbents

**Figure 3:**
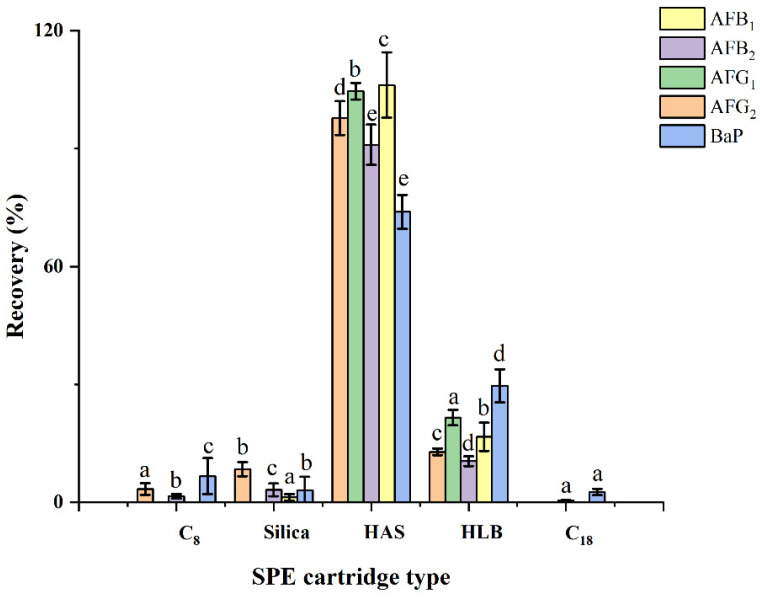
Effect of the types of SPE adsorbents on the recoveries of AFs and BaP. Data were analyzed using one-way ANOVA with Duncan’s multiple range test, and classified as group a, b, c, d and e.

To ensure sufficient recoveries of AFs and BaP, various types of SPE adsorbents were carefully evaluated. As shown in Figure 3, the maximum recoveries of AFs and BaP (73.9% to 106.2%) were achieved using HAS adsorbents. Conversely, the recoveries of the other four classical (C_8_, C_18_, and silica) and mixed-mode (HLB) SPE adsorbents showed poor recoveries (less than 30%), which did not fully satisfy the requirement for routine analysis. Due to various functional groups, HAS adsorbent captures both BaP and AFs from complex lipid matrices via hydrogen bonding, hydrophobic, and π–π interactions. Therefore, HAS adsorbents were selected to extract and purify AFs and BaP.

#### 2.2.3. Washing Solvent

**Figure 4:**
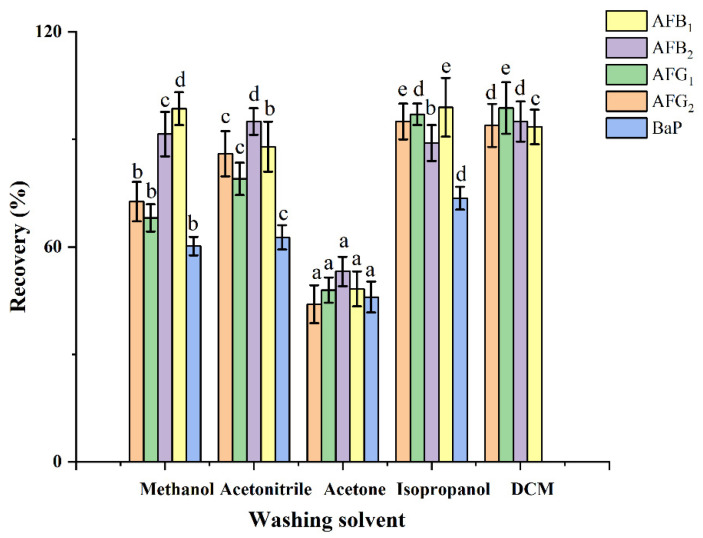
Effect of washing solvent on the recoveries of AFs and BaP. Data were analyzed using one-way ANOVA with Duncan’s multiple range test, and classified as group a, b, c, d and e.

To eliminate matrix interference and achieve excellent HPLC–FLD chromatography, the oil matrices and lipid-soluble compounds that were retained on the HAS adsorbent should be removed as much as possible. Figure 4 presents the recoveries of AFs and BaP achieved with several washing solvents, including methanol, acetonitrile, acetone, isopropanol, and dichloromethane. The highest recoveries were obtained by isopropanol, in the range from 73.6% to 99.0%. Therefore, 10 mL of isopropanol was selected as the washing solvent.

#### 2.2.4. Eluting Solvent

**Figure 5:**
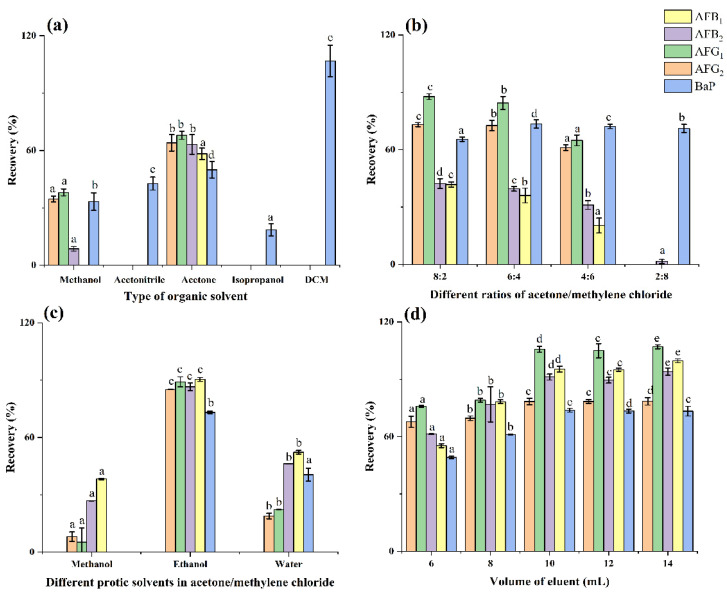
Optimization of eluting solvent: (**a**) type of organic solvent; (**b**) different ratios of acetone/methylene chloride; (**c**) different protic solvents in acetone/methylene chloride; (**d**) volume of eluent. The blank soybean oils were spiked with AFs and BaP at 5 μg kg^−1^. Data were analyzed using one-way ANOVA with Duncan’s multiple range test, and classified as group a, b, c, d and e.

To elute AFs and BaP from HAS–SPE, five solutions of different polarities (methanol, acetonitrile, acetone, isopropanol, and methylene chloride) were used, respectively. As illustrated in Figure 5a, pure solvents did not achieve acceptable recoveries of all analytes, which was attributed to the numerous types of interaction forces that exist between the analytes and HAS adsorbent. Therefore, a binary mixture of organic solvents (acetone/methylene chloride) was used as the eluting solvent. Figure 5b depicted the eluting efficiencies of AFs were greatly improved by the use of acetone/methylene chloride, and nearly 36–85% recoveries of AFs were obtained using 9 mL of the mixture. This mixture of aprotic solvents can interrupt most hydrophobic interactions between AFs and Bap with the HAS adsorbent. To further optimize the eluent, 1 mL protic solvents (methanol, ethanol, and water) were added into the aprotic mixture. Figure 5c indicated higher recoveries were obtained when ethanol was selected as the protonation solvent. Afterward, the volume of eluent, ranging from 6 to 14 mL, was optimized, as shown in Figure 5d. A volume of 10 mL eluent achieved the highest efficiencies (73.8% to 105.7%) for AFs and BaP. Finally, 10 mL of acetone/methylene chloride/ethanol (5.4/3.6/1, *v*/*v*/*v*) was used in the following experiments.

### 2.3. Method Validation

#### 2.3.1. Matrix Effect

By comparing the calibration curves, the slopes for the blank matrix fortified with standard solution (*Slope _matrix_*_+ *std*_) and the pure standard solutions (*Slope _std_*) were calculated. Table 1 showed the matrix effect (ME) on AFs and BaP analyses ranged from −19.5% to −8.3%, which indicates a slight signal suppression. No other matrix effects were explored in rapeseed, peanut, sunflower, corn, and blended oils. The HAS–SPE procedure effectively removed the lipid interferences and accurately isolated the target compounds from vegetable oils. Moreover, the pure solvent calibrations were used to quantify the concentration of analytes.

#### 2.3.2. Linearity and Sensitivity

The analytical parameters of the HAS–SPE HPLC–PHRED–FLD method were evaluated under the optimized conditions. In Table 1, all of the compounds achieved good linearity in the concentration range, and the correlation coefficients (*R*^2^) for all of the compounds were between 0.9913 and 0.9998. The sensitivity of the method was assessed by evaluating the LODs and LOQs of the spiked blank soybean oils, which were determined as the concentrations corresponding to signal-to-noise ratios of 3 and 10, respectively. The LODs and LOQs for AFs and BaP were in the range from 0.01 to 0.09 µg kg^−1^ and from 0. 05 to 0.30 µg kg^−1^, respectively.

#### 2.3.3. Accuracy and Reproducibility

The accuracy of the proposed method was evaluated as trueness (system error) and equated as the mean recoveries. Rapeseed oil and peanut oil samples were spiked at four levels, ranging from 1 to 20 μg kg^−1^. As shown in Table 2, the recoveries ranged from 66.9% to 118.4% in rapeseed oil and from 69.3% to 116.3% in peanut oil. Additionally, the intra-day and inter-day RSDs were less than 7.2%, respectively. The results indicated that the accuracy and reproducibility were acceptable.

### 2.4. Analysis of AFs and BaP in Vegetable Oils

**Figure 6:**
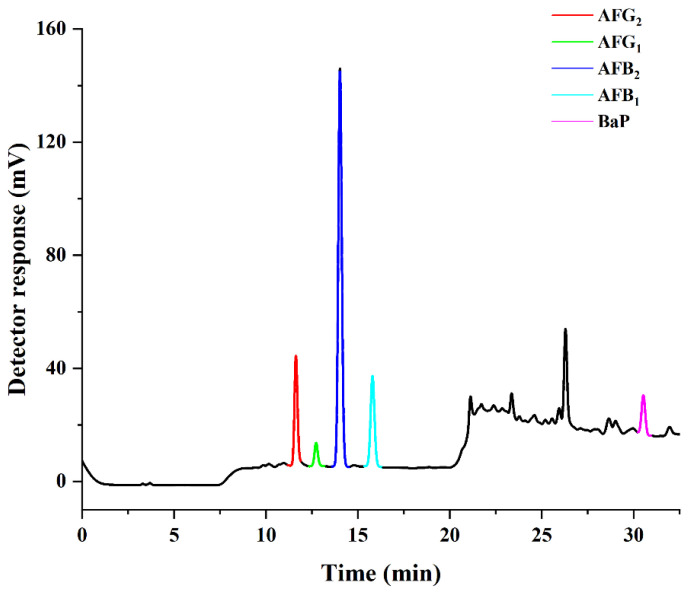
Typical HPLC chromatograms of AFs and BaP spiked at 4 μg kg^−1^ in soybean oil.

To evaluate the suitability of the proposed method for the multi-residue determination of AFs and BaP, 80 vegetable oils from local supermarkets were purchased, and three replicates were analyzed for each sample. As summarized in Table 3, the detection rates of AFs and BaP in the oil samples were notable. In peanut oil samples, the detection rate of AFB_1_ was 10%. For BaP, the detection rates of various oils were more than 70%. In all samples, 1.25% and 82.5% of analyzed oil samples were contaminated with AFB_1_ (0.30 µg kg^−1^) and BaP, ranging from 0.44 to 3.18 µg kg^−1^, respectively. However, the high occurrence of BaP does not necessarily suggest that additional quality and safety monitoring of vegetable oils is required, as most of concentrations were all within the MRL of EU and China food safety regulations. Figure 6 presented the HPLC–PHRED–FLD chromatogram of the blank soybean oil spiked with AFs and BaP at a concentration of 4 μg kg^−1^. The chromatogram showed sharp, narrow peaks with baseline resolution, indicating good adsorption of the HAS–SPE adsorbent for the target compounds and minimal matrix interference. The results indicated that the developed method can be used to accurately quantify trace amounts of AFs and BaP in oil samples with no interference from liposoluble substances.

A comparison of the HAS–SPE HPLC–PHRED–FLD method for the quantification of AFs and BaP in oils to previously reported methods was presented in Table 4. Because of the various functional groups, HAS adsorbent simultaneously retained both BaP and AFs from lipid matrices via hydrogen bonding, hydrophobic, and π–π interaction. When coupled with an online post-column photolysis PHRED–FLD analytic system, satisfactory LODs, RSDs, and recoveries were achieved in different types of vegetable oils. To date, only gel permeation chromatography HPLC–FLD has been used to extract and quantify both AFs and BaP in vegetable oils [29]. Compared with the instrumental purification, the whole time for SPE protocol was only approximately 30 min with simple procedure, which avoided the tedious steps and minimized pretreatment time and the volume of organic solvents. Additionally, the sensitivity of the method was comparable to that of MS or MS/MS, which can only analyze BaP in high concentrations. The results suggested that the proposed method was the first to use HAS adsorbents coupled with HPLC–PHRED–FLD, and it can be used to effectively quantify and evaluate AFs and BaP in vegetable oils.

## 3. Conclusions

In this work, we proposed a simple, reliable, and cost-effective HAS–SPE HPLC–PHRED–FLD method to quantify AFB_1_, AFB_2_, AFG_1_, AFG_2_, and BaP in vegetable oils. Under the optimized conditions, HAS–SPE can effectively separate the analytes from the lipid matrix in a large batch, without a labor-intensive preparation procedure. The recoveries ranged from 66.9% to 118.4%, with intra-day and inter-day precision less than 7.2%. Using the optimized conditions, trace levels of AFB_1_ and BaP were detected in a variety of vegetable oils. To our knowledge, this study represents the first report on simultaneous determination of AFs and BaP in oils using HAS–SPE HPLC–PHRED–FLD. The LODs of the analytes ranged from 0.01 to 0.09 µg kg^−1^, which meets the regulatory levels enforced by the EU and China. In general, HAS–SPE coupled with HPLC–PHRED–FLD was established as a novel technique for the simultaneous detection of AFB_1_, AFB_2_, AFG_1_, AFG_2_, and BaP in complex matrices.

## 4. Materials and Methods

### 4.1. Chemicals

HPLC-grade methanol, acetonitrile (ACN), and formic acid were purchased from Fisher Scientific (St. Louis, MO, USA). Ultra-pure water (18 mΩ) was obtained from a Milli-Q water purification system from Millipore Co., Ltd. (Milford, CT, USA). Humic acid-bound silica solid-phase extraction (HAS–SPE) cartridges were purchased from Weltech Co., Ltd. (Wuhan, China).

### 4.2. Standards

AFB_1_, AFB_2_, AFG_1_, AFG_2_, and BaP with ≥98% purity were purchased from Sigma-Aldrich Co., Ltd. (Shanghai, China). Stock solutions were freshly prepared for all of the standards by accurately weighing 5 ± 0.1 mg of each standard and dissolving it separately in 1 mL of acetonitrile. The mixed standards of AFs and BaP standard stock solutions were prepared in acetonitrile at 20 μg mL^−1^, then the working standard solutions were prepared from the above stock solutions with acetonitrile. All of the standard solutions were sealed with parafilm, covered with aluminum foil, and stored in the dark at 4 °C until use.

### 4.3. Preparation of Vegetable Oils

To further optimize and validate the HAS–SPE HPLC–PHRED–FLD method, vegetable oil samples were collected from local supermarkets, including blended oils (10), camellia oil (10), rapeseed oil (10), peanut oil (10), sunflower oil (10), corn oil (10), soybean oil (10), and sesame oil (10). According to the labeling information of vegetable oil products, the rapeseed and soybean oilseeds used for oil processing were non-genetically modified organism (GMO) materials. All of the vegetable oils were kept in the dark at 25 °C prior to analysis. The oils were mixed thoroughly by a mechanical mortar for 15 min, and immediately detected by the optimized method in triplicate.

### 4.4. HAS–SPE Adsorbents and Procedure

HAS–SPE adsorbents were sequentially preconditioned with 5 mL of acetone/water (8/2, *v*/*v*) and 5 mL of *n*-hexane. Two grams of oil was precisely weighed into a 15 mL centrifugal tube, diluted with 8 mL of *n*-hexane, and vortexed for 10 s. Then, the diluted extract of oil solution was loaded onto the HAS–SPE cartridge. Then, the adsorbents were washed with 10 mL of isopropanol, and the analytes were eluted with 10 mL of acetone/methylene chloride/ethanol (5.4/3.6/1, *v*/*v*/*v*). The desorption solution was collected and evaporated to dryness under a mild nitrogen stream at 40 °C. The residue was re-dissolved with 200 μL of acetonitrile and subjected to HPLC–PHRED–FLD analysis.

### 4.5. HPLC–PHRED–FLD Analysis

Chromatographic analysis was conducted using the Wooking K2025 system from Shandong Wooking Technology Co., Ltd. (DeZhou, China) coupled with an online degasser, a binary pump, and photochemical post-column reactors (PHRED) with an ultraviolet lamp (k = 254 nm) and a knitted reactor coil from Wuhan Trustworthy Technology Co., Ltd. (Wuhan, China). HPLC chromatographic separation was performed at 35 °C on a Capcell Pak C_18_ column (4.6 mm × 150 mm, 5 μm). Mobile phase A consisted of methanol, and mobile phase B consisted of aqueous 0.05% formic acid. A linear gradient elution program was applied as follows: 0 min, 10% A, 4 min, 45% A, 17 min, 88% A, and 30–33 min, 10% A. Excitation and emission wavelengths for AFs and BaP were 380 and 420 nm, respectively. The flow rate was 1 mL min^−1^, and the sample injection volume was 10 μL.

### 4.6. Method Validation

The HAS–SPE HPLC–PHRED–FLD method was validated according to European Commission Decision 2002/657/EC and JRC Technical Reports [34,35]. The analytic parameters were studied including matrix effects, linearity, the limit of detection (LOD), the limit of quantification (LOQ), accuracy, and intra-day and inter-day precision.

#### 4.6.1. Matrix Effect

Pure solvents and blank samples were used to evaluate the matrix effect using the following equation:

(1) $$Matrix\;effect\;\left(\%\right)=\left[\frac{Slope_{\left(matrix\;+\;std\right)}}{Slope_{\left(std\right)}}-1\right]\times 100\%.$$

During HPLC–PHRED–FLD analysis, a ME less than 0% was categorized as ion suppression, and a ME more than 0% was categorized as ion enhancement. When the ME was not in the range of −20% to 20%, matrix-matched calibration was required to quantify the analytes in the oil samples.

#### 4.6.2. Sensitivity and Linearity

The LODs and LOQs were evaluated as the minimum quantifiable concentrations of AFs and BaP in the spiked samples of vegetable oil. The LODs were defined as the lowest detectable concentration with a signal-to-noise ratio (S/N) of 3, and the LOQs were defined as the lowest quantified concentration with S/N of 10, respectively.

The calibration study was performed in triplicate using blank soybean oil samples spiked at different concentrations of AFB_1_, AFB_2_, and AFG_2_ (0.1, 0.5, 1.0, 5.0, 10, 25, and 50 μg kg^−1^), AFG_1_ (0.3, 1.0, 5.0, 10, 25, and 50 μg kg^−1^), and BaP (0.05, 0.5,1.0, 5.0, 10, 25, and 50 μg kg^−1^), respectively. All of the calibration curves were constructed by calculating the chromatographic peaks of analytes via the corresponding concentrations.

#### 4.6.3. Accuracy and Precision

The accuracy of the HAS–SPE HPLC–PHRED–FLD method was evaluated by trueness (systematic error) and expressed in terms of the mean recovery. Blank rapeseed–peanut blend samples were spiked with different concentrations of AFs and BaP (1, 5, 10, and 20 µg kg^−1^, respectively). Sequentially, the spiked samples were used to evaluate the recoveries of AFB_1_, AFB_2_, AFG_1_, AFG_2_, and BaP, and the mean concentrations in the oil samples were calculated accordingly.

The reproducibility of the HAS–SPE HPLC–PHRED–FLD method was assessed through intra-day and inter-day precision analysis of the blended oils spiked with AFB_1_, AFB_2_, AFG_1_, AFG_2_, and BaP at 5 µg kg^−1^. The intra-day precisions were evaluated by the analysis of three parallel spiked samples within one day, and the inter-day precisions were evaluated by the analysis of six parallel spiked samples over six consecutive days.

### 4.7. Statistical Analysis

All of the samples were analyzed in triplicate, and the data were calculated as the average ± standard deviation (SD). Statistical analysis was performed using the SPSS statistics 22 (SPSS Inc., Chicago, IL, USA). Significant differences were determined by one-way analysis of variance (ANOVA) with Duncan’s multiple-range test at *p* < 0.05.

## Figures and Tables

**Table 1:** The linear range, calibration curve, limit of detection (LOD), limit of quantification (LOQ), pKa and ME for AFs and BaP.

Analytes	Matrices	Linear Range (μg kg^−1^)	LOD (μg kg^−1^)	LOQ (μg kg^−1^)	Regression Equation	*R* ^2^	ME (%)	pKa ^a^
AFB_1_	Solvent	0.10–50	0.03	0.10	y = 17.505x − 2.0209	0.9982	-	17.79
	Soybean oil	0.10–50	0.03	0.10	y = 15.137x − 1.2543	0.9984	−13.5	
AFB_2_	Solvent	0.10–50	0.03	0.10	y = 15.754x − 1.233	0.9993	-	17.79
	Soybean oil	0.10–50	0.03	0.10	y = 14.448x − 2.628	0.9991	−8.3	
AFG_1_	Solvent	0.30–50	0.09	0.30	y = 4.294x − 0.5869	0.9998	-	-
	Soybean oil	0.30–50	0.09	0.30	y = 3.514x − 0.5094	0.9933	−18.2	
AFG_2_	Solvent	0.10–50	0.03	0.10	y = 18.165x − 3.2121	0.9981	-	-
	Soybean oil	0.10–50	0.03	0.10	y = 15.412x − 15.839	0.9976	−15.2	
BaP	Solvent	0.05–50	0.01	0.05	y = 35.435x − 16.264	0.9977	-	-
	Soybean oil	0.05–50	0.01	0.05	y = 28.532x + 4.2851	0.9913	−19.5	

^a^ pKa value was confirmed as the strong acidic group from the online web of Toxin and Toxin Target Database (T3DB, http://www.t3db.org (accessed on 15 March 2022)).

**Table 2:** Recoveries and precisions of the HAS–SPE HPLC–PHRED–FLD method.

Analytes	Recovery (%, *n* = 6) ^a^								Precision (RSD, %) ^b^	
	Rapeseed Oil				Peanut Oil				Intra-Day (*n* = 6)	Intra-Day (*n* = 6)
	1 µg kg^−1^	5µg kg^−1^	10 µg kg^−1^	20 µg kg^−1^	1 µg kg^−1^	5 µg kg^−1^	10 µg kg^−1^	20 µg kg^−1^		
AFB1	76.5	85.3	101.6	96.9	83.3	98.1	98.8	100.2	1.7	2.5
AFB2	102.2	96.4	100.1	97.7	116.3	104.7	99.8	99.6	2.4	3.4
AFG1	114.8	89.4	106.3	97.7	102.8	95.3	101.0	99.9	3.8	7.2
AFG2	118.4	87.9	102.3	96.8	109.4	94.8	100.6	99.6	6.6	5.9
BaP	74.1	66.9	74.1	74.8	75.8	70.9	69.3	70.6	3.4	3.0

^a^ Recoveries, intra-day and inter-day precisions were investigated as the mean value in sextuplicate analysis. ^b^ Precisions of AFs and BaP were evaluated at 5 µg kg^−1^ in blank blend oil samples by calculating the RSDs.

**Table 3:** Results for the determination of AFs and BaP in vegetable oil samples ^a^.

Vegetable Oils	Number of Samples	AFs Content (μg kg^−1^)				Detection Rate (%)	BaP Content (μg kg^−1^)			Detection Rate (%)
		AFB_1_	AFB_2_	AFG_1_	AFG_2_		Max	Min	Mean	
Peanut oil	10	0.30	ND ^b^	ND	ND	10%	2.07	ND	0.99	90%
Corn oil	10	ND	ND	ND	ND	—	0.80	ND	0.45	70%
Rapeseed oil	10	ND	ND	ND	ND	—	0.88	ND	0.41	70%
Blended oil	10	ND	ND	ND	ND	—	1.77	ND	0.99	90%
Camellia oil	10	ND	ND	ND	ND	—	1.28	ND	0.69	80%
Soybean oil	10	ND	ND	ND	ND	—	2.34	0.44	1.15	100%
Sesame oil	10	ND	ND	ND	ND	—	1.33	ND	0.71	80%
Sunflower oil	10	ND	ND	ND	ND	—	3.18	ND	1.82	80%

^a^ The content of analyte was investigated as the mean value in triplicate analysis. ^b^ ND, no detect.

**Table 4:** Comparison of sample preparation procedures and LOQs with different methods.

Sample	Analytes	Pretreatment	Determination Technique	LOQs (µg kg^−1^)	Advantages and Drawbacks	Ref.
Vegetable oil	BaP	HAS–SPE	HPLC–FLD	0.2	Simple, rapid, and high sensitivity but detects one class of analyte	[27]
Vegetable oil	BaP	Supramolecular solvent microextraction	HPLC–FLD	0.19	Uses less amount of organic solvent but requires tedious step including saponification and centrifugation	[31]
Coix seed	AFB_1_, AFB_2_, AFG_1_, AFG_2,_ ZON, α-ZOL, β-ZOL	IAC	HPLC–PCD–FLD	0.04~0.32	Suitable and high-throughput but the immune adsorbents are expensive and not available for BaP	[32]
Cereal crop	AFB_1_, AFB_2_, AFG_1_, AFG_2_	SPE	HPLC–FLD	0.3~1	Simple and rapid but involving complicated steps for synthetic adsorbents and pre-column derivatization	[33]
Vegetable oil	AFB_1_, AFB_2_, AFG_1_, AFG_2_	HAS–SPE	LC–MS/MS	0.039~0.12	Rapid and high sensitivity but needs sophisticated apparatus	[28]
Vegetable oil	AFB_1_, AFB_2_, AFG_1_, AFG_2_, BaP	GPC	HPLC–FLD	1.66~3.33	Automated pre-processing but involving tedious steps and preparation equipment	[29]
Vegetable oil	AFB_1_, AFB_2_, AFG_1_, AFG_2_, BaP	HAS–SPE	HPLC–PHRED–FLD	0.05~0.3	Simple, rapid, high sensitivity, cost-effective and multi-residue determination	This work

## Data Availability

Not applicable.
